# Attachment in the time of COVID-19: Insecure attachment orientations are associated with defiance of authorities’ guidelines during the pandemic

**DOI:** 10.1177/02654075221082602

**Published:** 2022-08

**Authors:** Joel Gruneau Brulin, Phillip R. Shaver, Mario Mikulincer, Pehr Granqvist

**Affiliations:** 1 7675 Stockholm University, Stockholm, Sweden; 2 University of California, Davis, CA, USA; 3 Baruch Ivcher School of Psychology, Reichman University (IDC Herzliya), Israel

**Keywords:** attachment, COVID-19, emotion, stress and coping, trust

## Abstract

Previous research has linked people’s relational attachment orientations to emotional reactions and coping during crises, and to social trust and trust in societal institutions. The COVID-19 pandemic is a global crisis for which collective efforts, such as social distancing, are necessary to stop the spread of the virus. During previous pandemics, people high in trust have typically adhered to such efforts. In the present study, we investigated whether attachment orientations were related to people’s adherence to authorities’ guidelines to stop the spread of COVID-19. We also tested whether previous mediational findings—linking attachment-related avoidance to welfare state trust via social trust—would replicate. We used a web-based survey of 620 participants. Our findings showed that attachment-related anxiety was linked to *low* adherence to social distancing regulations. This finding was especially noteworthy because high attachment-anxious participants also experienced more negative emotions, yet negative emotions were generally linked to *high* adherence. Thus, people higher in attachment anxiety seem to have more difficulties in avoiding social situations despite heightened risk of catching and spreading the virus. In addition, attachment-related avoidance was negatively related to adherence and to welfare state trust, and its effects on welfare state trust were statistically mediated by low social trust.

## Introduction

At the beginning of 2020, the corona virus started to spread rapidly across the world. By mid-March, the World Health Organization declared it a pandemic (World Health Organization, 2020, March 11). In the ensuing weeks, governments in country after country tried to prevent the spread of the virus through major restrictions for businesses and on people’s mobility. Airline traffic was stopped, public schools closed down, many employees were forced to work from home, and many other lockdown-related measures were taken. Different nations employed different restrictions, relying to varying degrees on regulations, fines, and behavioral guidelines to assure that the populations maintained social distance. For example, in Spain, China, and Italy, nationwide lockdowns were employed, including closure of schools and imposing fines for moving about too far from home. In other countries, including the United Kingdom in the beginning, and Sweden, governments relied on more liberal approaches, keeping large parts of society open, but employing social distancing guidelines to prevent catching and spreading the virus. National public health authorities’ interpretation of the scientific evidence-base was central for these guidelines.

Trust is a key factor affecting people’s adherence to authorities’ regulations and guidelines (Bargain & Aminjonov, 2020), and arguably even more so when authorities rely on a more liberal approach (e.g., guidelines vs. regulations) to stop the spread of a virus (as noted by Dorit Nitzan, the WHO’s regional emergency director for Europe [Henley, 2020]). In these cases, if non-compliance is not penalized, compliance must be secured by some other means, such as mutual trust. Firstly, the authorities need to trust that the population at large will actually follow the guidelines. Secondly, the population (again at large) needs to trust that the government has the population’s best interests in mind. Thirdly, population members need to trust one another—since social distancing is effective only if most members of a population engage in it, people need to trust that others will also adhere to the guidelines.

According to attachment theory trust is developed through contingent experiences of loving and caring others in early close relationships (Bosmans et al., 2019; Bowlby, 1973). Such experiences are internalized as internal working models (IWMs) which function as relational predictors, including predictions about other’s trustworthiness in future relationships. In a previous article (Gruneau Brulin et al.), we found that attachment working models (specifically attachment-related avoidance) expand beyond trust in close relationships and was also related to lower trust in people in general, as well as to public authorities such as the welfare state. As trust is an important predictor of adherence of authorities’ guidelines to prevent the spread of the virus (Bargain & Aminjonov, 2020; Han et al., 2021), it may be that attachment orientations have relevance beyond close relationships, and possibly could have an effect on societal behavior, such as adhering to the guidelines. In the present study, we therefore extended our previous findings by investigating whether attachment orientations (anxiety, avoidance) in close relationships are related not only to trust but also to adherence to authorities’ social distancing guidelines for stopping the spread of COVID-19. In addition, we explored whether attachment insecurities make a unique contribution to adherence over and above that of negative emotions and trust. Specifically, we investigated this in Sweden, a country where the government adopted one of the most liberal approaches worldwide, including keeping society largely open and instead relying on a number of behavioral guidelines provided by the public health authority.

## Attachment theory and research

Attachment theory postulates that people are born with an innate behavioral and motivational system that is activated in stressful situations and motivates a person to strive for proximity with close others when in distress (Cassidy & Shaver, 2016). Originally, attachment theory was developed to characterize and explain the relationships between infants and their caregivers (Bowlby, 1969/1982), but it was later extended and applied to adult close relationships and emotional wellbeing (Hazan & Shaver, 1987). The theory states that experiences in close relationships result in the creation and elaboration of internal working models (IWMs), or interpersonal expectancies, that influence personality development, emotion regulation, and perception of trustworthiness of others in future relationships (Bowlby, 1973). Although Bowlby focused primarily on close relationships, he also suggested that attachment experiences have an impact on a person’s perceptions of and behavior outside of close relationships, of the world at large.

Depending on experiences in close relationships, especially in the context of stressful or frightening situations, people develop a persisting sense of attachment security or one of several possible insecure patterns of attachment (Ainsworth et al., 1978). Attachment security is characterized by beliefs that others are trustworthy and will generally be supportive, that one is generally lovable, and that it is possible to be both support-seeking, when support is needed, and self-reliant and helpful to others when support for oneself is not needed (Mikulincer & Shaver, 2016). Insecure patterns in adulthood can be conceptualized in terms of two orthogonal dimensions: attachment-related avoidance and anxiety, where low scores on both dimensions indicate a greater degree of attachment security (Brennan et al., 1998).

## Attachment orientations

Attachment-related avoidance is characterized by negative representations of others, discomfort with closeness in relationships, and by denial and suppression of attachment-related needs (Mikulincer & Shaver, 2016). Individuals higher in avoidance tend to deactivate their attachment system, even in distressing situations, thus directing their attention away both from the trigger of the distress and away from other people. They are thus less inclined to seek support from others (Holmberg et al., 2011) and more likely to rely on coping strategies that involve cognitive and social distancing as well as emotional disengagement (Mikulincer & Florian, 1998; Mikulincer & Shaver, 2016; Simpson & Rholes, 2017). Consequently, attachment-related avoidance is related to lower levels of reported emotional arousal in stressful situations, despite heightened arousal indicated by psychophysiological measures such as blood pressure recovery (Ehrenthal et al., 2011) and heart rate variability (Maunder et al., 2006). Notably though, previous research also suggests that attachment-related avoidance is related to faster action-taking in some stressful situations, presumably because of self-interest and lower acknowledgment of threat (Ein-Dor et al., 2011b).

Attachment-related anxiety, on the other hand, is characterized by hyperactivation of attachment needs and behaviors, marked by excessive worrying about one’s lovability, fearing abandonment, being sensitive to possible rejection and highly emotional in response to separation and loss (Mikulincer & Shaver, 2016). In stressful situations, attachment anxiety has been related to greater distress and more negative emotions (Shallcross et al., 2014), and to usage of emotional coping strategies such as worrying and rumination (Caldwell & Shaver, 2012). Attachment anxiety is also related to greater distress when alone (Mikulincer et al., 2021). Characteristically then, when in distress individuals higher in attachment anxiety both experience greater degree of distress and a stronger urge to be close to others (Mikulincer et al., 2003). Moreover, attachment anxiety is related to quicker detection of threats (Ein-Dor et al., 2011a), although typically individuals higher in attachment anxiety take longer to act in response to a threat. It seems that their attentional focus and emotional reactions remain inflexibly fixated on the threat and on their perception of potential negative consequences, which interfere with constructive problem solving (Silva et al., 2012).

Global crises, like the COVID-19 pandemic, trigger worries and distress (e.g., Taylor et al., 2020), thus presumably activating the attachment system (Steele, 2020), and attachment orientations should then become relevant in explaining individual differences in emotional reactions and behaviors to handle these reactions. Previous studies have yielded supportive evidence. For example, attachment anxiety has been related to greater distress during the pandemic (Mazza et al., 2021; Moccia et al., 2020) and to more effort in convincing others to take precautionary actions, such as wearing face masks or washing hands (Lozano & Fraley, 2021). Attachment-related avoidance, on the other hand, has been linked to lower distress (Mazza et al., 2021; Moccia et al., 2020) and less inclination to take precautionary actions (Lozano & Fraley, 2021). However, these studies have mainly focused on links between attachment variations and emotional reactions, and have overlooked the more proximal role of representations of others, for example, the perceived trustworthiness of others, and how this may impact behavior during the pandemic. Since trust has previously been shown an important predictor of precautionary actions during pandemics (e.g., Prati et al., 2011; Velan et al., 2011), and because trust is a central aspect of one’s IWMs (Mikulincer, 1998), attachment orientations might affect behavioral reactions to the pandemic not only via reactions to distress but also via trust.

### Attachment and trust

Trust is typically divided into social and political trust (Newton et al., 2018). Social trust refers to one’s confidence or faith in other people, either people in general with whom one has no prior relationship (generalized social trust), or particular relationships, such as one’s partner, friends, or people in one’s community (particular trust). Political trust on the other hand refers to one’s confidence in political institutions, such as the government, judicial system, or welfare state institutions.

According to attachment theory, trust is part of one’s internal representations of others (i.e., IWMs) and is shaped through contingent experiences of others in close relationships (Bosmans et al., 2019; Bowlby, 1973; Mikulincer, 1998). Accordingly, previous research shows that attachment security is indeed related to higher degree of trust in close, dyadic relationships, including romantic ones (Campbell & Stanton, 2019; Simpson, 2007). For example, attachment insecurity—both avoidance and anxiety—is negatively related to trust in romantic partners (Collins & Read, 1990; Simpson, 1990), and this lower trust is in turn related to lower relationship satisfaction (Fitzpatrick & Lafontaine, 2017). Attachment security has also been linked to faster mental access to trust-related cognitions and memories and to higher expectations of partners to be trustworthy (Mikulincer, 1998). This suggests that individuals higher in attachment security may possess IWMs of others as more trustworthy and hence find it easier to both place their confidence in others, and perceive behaviors of others as more trustworthy. However, research is scant on whether this trust extends beyond a particular close relationship, to impact also a person’s expectations and perceptions regarding people more generally (generalized trust), or in relation to non-human entities such as political institutions.

Bowlby hypothesized that attachment working models do not only impact close relationships but also how one perceives and relates to the world at large (Bowlby, 1973). Attachment working models (IWMs), formed through experiences in close relationships, may then expand and influence people’s perception of others outside of close relationship as well. In line with this idea, a hierarchical structure of IWMs comprising both specific attachment models of particular relationships and global overarching models for relationships have demonstrated better fit compared to a structure containing only relationship-specific IWMs (Overall et al., 2003). Relatedly, one’s image of non-human entities such as God are coherent with one’s IWM of other people, for example, secure people perceive God as more loving (Granqvist, 2020).

Regarding specifically trust, (Bradshaw et al., 2019) found that individuals with higher attachment security to God expressed higher trust in people in general and in specific relationships, and (Gillath et al., 2020) found that trust in artificial intelligence was negatively related to attachment anxiety. In a previous study, we also found that attachment-related avoidance (but not attachment anxiety) was associated with lower levels of generalized social trust and with trust in political institutions (i.e., trust in welfare state institutions, (Gruneau Brulin et al.). Furthermore, the relation between avoidance and welfare state trust was statistically mediated by social trust, suggesting that attachment-related avoidance has an impact on one’s perception of the trustworthiness of people in general and that this social trust generalizes or extends to trust in political institutions. Hence, attachment orientations presumably have important implications not only for particular trust in dyadic relationships, but also for trust in people in general and in political institutions, thus presumably influencing large-scale societal interactions and systems, for example, during a pandemic.

### Trust and adherence to guidelines

Arguably, particular and generalized social trust have different implications for social interactions (Igarashi et al., 2008). Particular trust regards trust when one already has an established connection with the other, while generalized trust regards trust in people one does not know. The latter is of key importance for societal behavior and large-scale cooperation, as this involves cooperation with people one does not know (Van Lange, 2015). Countries with higher levels of generalized social trust report lower levels of corruption (Rothstein & Uslaner, 2005), greater willingness to pay taxes (Scholz & Lubell, 1998), and higher economic growth (Knack & Keefer, 1997). Also, country-level generalized social trust has been found to predict *later* (larger) size of a welfare state (Bergh & Bjørnskov, 2014).

During a pandemic trust may impact behavior and actions in two ways. Firstly, precautionary actions, mandated by authorities through official guidelines, are only effective if most people adhere to these guidelines, thus creating a large-scale social dilemma. Hence, trust in others—that others will adhere to the guidelines—would impact one’s own inclination to act in accordance with the guidelines. Secondly, trust in political institutions—that one has confidence in the competence of the authorities and that the authorities act in the populations’ best interest—would increase willingness to follow the authorities’ guidelines. Accordingly, during previous pandemics both generalized social trust and political trust have been associated with greater willingness to act in accordance with governmental regulations and to partake in societal programs to stop the spread of the disease (Prati et al., 2011; Velan et al., 2011). For example, during the H1N1 (“swine flu”) pandemic in 2011, a Swiss study found that political trust predicted vaccination status six months later (Gilles et al., 2011). A Swedish study similarly found that people reporting higher generalized social trust and trust in health organizations were more inclined to get vaccinated (Rönnerstrand, 2013). During the current COVID-19 pandemic, trust has been predictive of behavior that can mitigate the spread of the virus. For example, trust in politicians predicted less mobility—thus less virus spread—during the beginning of the pandemic in Europe (Bargain & Aminjonov, 2020). A large cross-cultural study similarly found that trust in government was positively related to pertinent safety behavior, such as maintaining social distance and washing hands (Han et al., 2021). Some studies also indicate that trust might have increased as a consequence of the pandemic crisis (Baekgaard et al., 2020; Sibley et al., 2020).

### The present study

The aim of the present study was twofold. The first aim was to extend our previous findings, showing that attachment orientations in close relationships are relevant beyond close relationships per se and are related to generalized social and political trust (Gruneau Brulin et al.) as well as other findings linking trust with people’s adherence to public health guidelines during pandemics. More specifically, in the present study, we examined whether attachment orientations relate to adherence to official guidelines intended to prevent the spread of COVID-19. Presuming such an association, we also asked whether generalized social and political trust statistically mediated the relation.

Because previous studies had shown that trust is positively related to adherence to official guidelines during pandemics (Rönnerstrand, 2013; Velan et al., 2011) and that attachment-related avoidance is negatively related to trust (Gruneau Brulin et al.), we hypothesized that avoidance would be negatively associated with adherence to the official guidelines. We also expected that trust (political trust in particular) would mediate this relation.

Regarding attachment anxiety, predictions were less straightforward. Attachment anxiety has been linked both with stronger negative emotional reactions in stressful situations (Shallcross et al., 2014) and with more distress during the present pandemic (Mazza et al., 2021; Moccia et al., 2020). Such distress reactions could propel stronger adherence to official guidelines to prevent catching the virus (Anaki & Sergay, 2021). However, individuals higher in attachment anxiety tend to constantly seek comfort and support from others, exaggerate dependence on relationship partners, and experience intense distress when alone. Therefore, attachment anxiety could also relate to defiance of social distancing guidelines and seeking of close contact with others. Due to these two competing possibilities, we refrained from making a specific prediction regarding the association between attachment anxiety and adherence. In addition, because insecure, particularly anxious, attachment has been associated with higher levels of negative emotion (Meyer et al., 2015) and with trait anxiety (Noftle & Shaver, 2006), and to differentiate between attachment anxiety and trait anxiety, we included both negative emotions and trait anxiety as covariates in the statistical analyses. Demographic factors (age, gender, educational level, income, and political orientation) were also included as covariates.

Our second aim was to replicate our previous findings (Gruneau Brulin et al.). Thus, we examined whether attachment-related avoidance was associated with political trust (trust in welfare state institutions) and whether this association was statistically mediated by social trust. The predictions here are self-evident. For pre-registration of the hypotheses, see https://osf.io/84796.

Importantly, our data were collected in Sweden, a country where the government applied one of the most liberal approaches worldwide to stop the spread of COVID-19. Instead of imposing and enforcing policies, the Swedish government relied on (i.e., trusted) people’s willingness to act in accordance with official guidelines.

## Method

### Participants

Data collection was performed online during the first wave of the COVID-19 pandemic in April and May 2020 in Sweden. Participants were mainly recruited through social media platforms such as Facebook. Due to an initial overrepresentation of women and people with higher education, male participants were targeted specifically through the participant recruitment platform studentkaninen.se and compensated with a lottery ticket (value approximately 3€). To participate in the study, participants had to be at least 18 years old and be residents of Sweden. No restrictions regarding place of residence in Sweden were made. Six hundred twenty participants finished the questionnaires (382 identified themselves as cisgender women, 232 as cisgender men, and six did not want to specify their gender), with the mean age of 41.36 (Mdn = 38, *SD* = 13.58, min = 18, max = 81). The vast majority of the participants (76%) were working (13% students, 7% retired, 4% unemployed, and 1% non-respondents), and 62% of the participants had a bachelor’s degree or higher. The ethical guidelines of Stockholm University and the American Psychological Association were followed.

### Measurements and procedure

The study consisted of a number of different questionnaires that were filled out online at the participants’ own pace. Participants were asked about their adherence to authorities’ guidelines to prevent the spread of COVID-19, followed by background socio-demographic questions (age, gender, educational level, occupation, income, and political orientation: left [1] to right [7]), and then questionnaires assessing attachment orientations, trust, negative emotions, and trait anxiety.

### Adherence to authorities’ guidelines

Seven items regarding adherence to guidelines intended to prevent the spread of COVID-19 were constructed based on the official guidelines provided by the Swedish public health authority (Folkhälsomyndigheten, 2020, April 20; see Appendix). The official guidelines were altered to form statements regarding the degree to which the participants had adhered to the statements during the past four weeks (e.g., “Stayed at home if you felt unwell,” scale 1 = “Not at all” to 7 = “Completely”). The seven items formed an internally consistent scale (α = .78), so the mean value for the seven items was used as the outcome variable in subsequent analyses. For descriptive statistics, see Table 1.

**Table 1.** Means, standard deviations, and bivariate Pearson’s correlation coefficients for variables included in the regression models.

Variable	Means (SD)	1	2	3	4	5	6	7	8	9	10	11	12	13
1. Adherence to guidelines	5.82 (1.08)													
2. Welfare state trust	4.55 (1.29)	.05												
3. Trust in FHM	5.64 (1.35)	.09*	.46**											
4. Social trust	5.08 (0.93)	.16**	.38**	.29**										
5. Attachment avoidance	2.95 (1.21)	−.09*	−.16**	−.08*	−.37**									
6. Attachment anxiety	3.25 (1.33)	−.16**	−.05	−.05	−.05	−.06								
7. Negative emotions	3.22 (1.58)	.20**	−.09*	−.08	.00	.04	.30**							
8. Trait anxiety	3.22 (1.46)	.01	−.15**	−.09*	−.05	.03	.41**	.50**						
9. Pol. Orientation (right)	3.01 (1.66)	−.17**	−.15**	−.25**	−.15**	.02	−.09*	−.17**	−.18**					
10. Knowledge of guidelines	3.01 (1.66)	.23**	.11**	.17**	.12**	−.11**	−.08*	.03	−.03	−.05				
11. Age	6.07 (1.05)	.39**	.10*	.17**	.10*	−.01	−.21**	-.05	−.20**	−.05	.09*			
12. Female	—	.32**	.06	.13**	.13**	−.15**	.03	.24**	.16**	−.26**	.21**	.24**		
13. Education	†	.14**	.19**	.09*	.07	−.06	−.03	−.00	−.00	−.20**	.19**	.06	.26**	
14. Income	30–40 000‡	.15**	.24**	.06	.11**	−.10**	−.14**	−.10*	−.22**	.10*	.18**	.29**	.08*	.39**

Note. * indicates *p* < .05. ** indicates *p* < .01. Political Orientation: higher values = right. FHM = Folkhälsomyndigheten (Public Health Authority).

† Education: 1 = high school, or less = 18%, 2 = some college = 25%, 3 = bachelor degree = 29%, 4 = master degree = 29%, 5 = doctoral degree 5%, no-response = 5%.

‡ Median income of yearly salary in €.

### Attachment orientations

To assess participants’ attachment orientations, a 12-item version of the Experiences in Close Relationships scale was used (Lafontaine et al., 2016; for the original, longer version see Brennan et al., 1998). Six items tap attachment-related avoidance (e.g., “I don’t feel comfortable opening up to romantic partners,” α = .81), and six items tap attachment anxiety (e.g., “I worry about being alone,” α = .82). Participants rated their agreement with each item, using a 7-point scale (1 = “Do not agree at all” to 7 = “Completely agree”).

### Trust

To assess political trust, we used the welfare state trust scale, which consists of eight statements regarding trust in different domains of the Swedish welfare state (e.g., “I trust publicly provided elder care in Sweden”) and in the welfare state in general (“I trust the social safety net”; scale 1 = “Do not agree at all” to 7 = “Completely agree”). The scale has previously exhibited good psychometric qualities (Gruneau Brulin et al.) and was satisfactorily internally consistent in the present study (α = .88). We also added two questions concerning participants’ trust in the public health authority and their knowledge of the guidelines provided by the public health authority.

Social trust was measured with the general social trust scale (Yamagishi & Yamagishi, 1994). It consists of six items concerning the trustworthiness of other people in general, rated on a 7-point scale (e.g., “Most people are trustworthy,” scale 1 = “Do not agree at all” to 7 = “Completely agree,” α = .84).

### Negative emotions

Negative emotionality was assessed with three items from the Positive and Negative Affect Scale (PANAS, (Watson et al., 1988), asking to what degree participants had experienced worry, anxiety, or fear during the past four weeks (scale 1 = “Not at all” to 7 = “Very much,” α = .88).

### Trait anxiety

Trait anxiety (one facet of neuroticism) was assessed with four items taken from IPIP-NEO (Maples et al., 2014), asking how characteristic it is for the participant to, for example, “Worry about things” (scale 1 = “Not at all” to 7 = “Very much,” α = .87).

## Results

### Adherence to the public health authorities’ guidelines

Our goal was to determine whether attachment-related avoidance and anxiety, as well as social and political trust, were related to adherence to the public health authority’s guidelines for reducing the spread of COVID-19. We therefore performed a 3-step hierarchical linear regression analysis with adherence to the guidelines as the outcome variable. In the first step, the covariates were entered as predictors: age, gender, education, income, political orientation (left-right), knowledge of the guidelines, negative emotions, and trait anxiety. In the second step, attachment-related avoidance and anxiety were entered as additional predictors and their unique contribution to adherence was examined. In the third and final step, we entered social trust, welfare state trust, and trust in the public health authority, examined their unique contribution to adherence, and determined whether the contribution of attachment anxiety and avoidance observed in Step 2 decreased after the introduction of the trust-related variables. This allowed us to test the impact of attachment anxiety and avoidance and to determine whether trust statistically mediated the presumed effects of attachment anxiety or avoidance.

We first present results regarding trust and the covariates before turning to the contribution of attachment orientations (for descriptive statistics and bivariate correlations, see Table 1).

In contrast with our hypothesis, the results revealed no significant effect of political trust on adherence to the guidelines (see Table 2). However, social trust had a significant but weak positive effect on adherence (see Table 2). In addition, whereas trust in the public health authority was not significantly related to reported adherence to the guidelines, knowledge of the guidelines made a significant unique contribution to adherence (see Table 2). These results indicate that it is not trust in the public health authority, or political trust, that drives adherence to the guidelines but rather knowledge about the guidelines and the perceived trustworthiness of other people. Moreover, older age, female gender, and more negative emotions were also significantly related to stricter adherence to the guidelines (see Table 2).

**Table 2.** Standardized beta coefficients for the regression models with adherence to authorities’ guidelines as outcome.

	Step 1		Step 2		Step 3	
Predictor	ß	CI (95%)	ß	CI (95%)	ß	CI (95%)
Age	0.33**	[0.25, 0.41]	0.31**	[0.24, 0.39]	0.31**	[0.24, 0.39]
Female	0.15**	[0.07, 0.23]	0.14**	[0.06, 0.22]	0.13**	[0.06, 0.21]
Education	0.04	[−0.04, 0.12]	0.04	[−0.04, 0.11]	0.04	[−0.04, 0.12]
Income	0.01	[−0.07, 0.09]	0.00	[−0.08, 0.08]	0.00	[−0.08, 0.09]
Pol. Orientation (right)	−0.07	[−0.14, 0.01]	−0.07*	[−0.14, −0.00]	−0.07	[−0.15, 0.00]
Knowledge of guidelines	0.15**	[0.08, 0.22]	0.13**	[0.06, 0.20]	0.13**	[0.06, 0.20]
Negative emotions	0.19**	[0.10, 0.27]	0.21**	[0.13, 0.29]	0.21**	[0.13, 0.29]
Trait anxiety	−0.06	[−0.15, 0.02]	−0.01	[−0.10, 0.07]	−0.01	[−0.10, 0.07]
Attachment avoidance			−0.08*	[−0.15, −0.01]	−0.05	[−0.13, 0.02]
Attachment anxiety			−0.16**	[−0.24, −0.09]	−0.16**	[−0.23, −0.08]
Welfare state trust					−0.05	[−0.13, 0.03]
Trust in FHM					−0.02	[−0.11, 0.06]
Social trust					0.08*	[0.00, 0.16]
Intercept	3.34**	[2.74, 3.94]	3.99**	[3.32, 4.66]	3.72**	[2.86, 4.58]
Adjusted R^2^	.264**	[.20, .31]	.289**	[.22, .33]	.295**	[.22, .34]
ΔR^2^			.025**	[.00, .05]	.006	[−.00, .02]

Note. Values in square brackets indicate the 95% confidence interval for each coefficient. * indicates *p* < .05, ** indicates *p* < .01. Political Orientation: higher values = right, FHM = Folkhälsomyndigheten (Public Health Authority).

Both attachment-related avoidance and anxiety were negatively related to adherence to the authorities’ guidelines (Δ*R*^*2*^ = .025). As hypothesized, attachment-related avoidance had a negative, though weak, effect on adherence (see Table 2). Because welfare state trust was unrelated to adherence, it did not, in contrast to our predictions, mediate the association between avoidant attachment and lower adherence. Instead, social trust appeared to act as a mediator (i.e., the effect of avoidance dropped to non-significance in step 3 of the regression analysis, following the inclusion of the significant contribution of social trust, see Table 2). To formally examine whether the relation between attachment-related avoidance and adherence was statistically mediated by social trust, we performed a path analysis with bootstrapping (Hayes, 2013). In this analysis, the same potential covariates were included. The mediation model could not fully confirm a mediational effect, however, because the confidence interval included zero (indirect effect: *ß* = −0.03, Bootstrap [*n* =10 000] CI 95% [−0.05, 0.00]).

Attachment anxiety also had a negative effect on adherence (see Table 2). Because attachment-related anxiety was unrelated to the trust variables, mediation by trust (social or political) was not tested. Interestingly however, participants with higher attachment anxiety scores also reported stronger negative emotions during the previous four weeks (*r* = .30, *p* < .001). As noted above, negative emotions were in turn related to higher (not lower) adherence. To follow up on these results, we also explored a possible interaction effect between attachment anxiety and negative emotions on adherence, but no significant interaction effect was found (see online materials for details: https://osf.io/rac6k). In other words, whether they scored high or low on negative emotions, highly anxious participants tended to defy the authority’s guidelines.

### Replication of the association between attachment orientations and political trust

Following our second goal, we examined whether our previous findings that attachment-related avoidance was associated with trust in the welfare state and that this association was statistically mediated by social trust would replicate in the present study. A hierarchical linear regression model with trust in the welfare state as the outcome was tested. First the covariates were entered (age, female gender, education, income, and political orientation), then attachment-related anxiety and avoidance, and finally social trust. The model confirmed the previous findings showing that avoidance was negatively related to trust in the welfare state (*ß* = −0.15, *p* < .001), but attachment anxiety was not (*ß* = −0.04, *p* = .294). To further test mediation, we performed a path analysis (Hayes, 2013) with bootstrapping (*n* = 10 000), which showed that social trust mediated the relation between avoidance and trust in the welfare state (indirect effect of avoidance on welfare state trust through social trust: *ß* = −0.13, Bootstrap CI 95% [−0.16, −0.09]). Thus, the results from our previous study were successfully replicated.

## Discussion

As in our previous study (Gruneau Brulin et al.), attachment-related avoidance was negatively associated with trust in the welfare state, and this relation was statistically mediated by social trust. Both attachment-related avoidance and anxiety were also negatively related to people’s adherence to public health authorities’ safety guidelines in relation to COVID-19. In addition, social trust was linked to lower adherence to the guidelines, but in contrast to our hypothesis, welfare state trust was not. Despite a descriptive indication that social trust partially mediated the effect of attachment-related avoidance on adherence, such mediation was not fully confirmed in a formal mediation analysis. Finally, attachment anxiety was related to more negative emotions. As in previous studies (e.g., Anakin & Sergay, 2021), negative emotions were related to higher degree of adherence to the public health authorities’ guidelines. However, despite individuals with higher attachment-anxiety reporting more negative emotions, they adhered to a *lesser* extent to the authorities’ guidelines.

### Trust and adherence

Previous studies investigating the relation between trust and cooperative behavior to prevent the spread of pandemics, typically through vaccination, have found positive links between cooperation and both political and generalized social trust (e.g., Rönnerstrand, 2013). In line with such findings, people with higher generalized social trust expressed greater adherence to the guidelines in the present study. This converges with the conceptualization of stopping the spread of the virus as a social dilemma; to get the maximum effect of vaccination or social distancing, a large proportion of the population needs to adhere to the guidelines. If a person trusts that other people will follow the guidelines, there should be a stronger inclination for that person also to do so. This effect remained significant even after controlling for pertinent covariates such as age, income, and educational level. Note, however, that when controlling for covariates, the effect of social trust was small (*ß* = .08, *p* = .038). Thus, the practical significance of this finding should not be overstated.

In contrast with previous studies, no effect of political trust, either trust in the welfare state or trust in the public health authority, was seen. However, the authorities in Sweden received a lot of criticism for not acting strongly enough to stop the spread of the virus and for not providing strong enough guidelines. It is therefore possible that some people practiced social distancing and followed other sensible practices regardless of the authorities’ guidelines. Hence, distrust in the authorities could have been due to disapproval of the comparatively mild (compared with other countries) precautions taken to stop the spread of COVID-19. Therefore, distrustful people might for example apply more social distancing and counterintuitively act more in accordance with the official guidelines, despite not trusting the authorities that provide the guidelines.

### Attachment avoidance, trust, and adherence

As in our previous study (Gruneau Brulin et al.), attachment-related avoidance was associated negatively with trust in the welfare state, a relation that was statistically mediated by generalized social trust. In contrast with our predictions, however, welfare state trust did not mediate the association between avoidance and adherence to the COVID guidelines, but generalized social trust did (at least marginally). Because highly avoidant people generally have more negative models of others (Mikulincer & Shaver, 2016), and consequently are less trusting (Bradshaw et al., 2019; Fitzpatrick & Lafontaine, 2017; Gruneau Brulin et al.), this distrust might make them less convinced that others are adhering to the guidelines and thus might make them less inclined to act in accordance with the guidelines themselves. It should be noted, however, that the effects of both attachment-related avoidance and social trust were rather small, and the mediation model could not be fully confirmed because the confidence intervals included zero. Hence, interpretation of these findings should be made with caution.

Previous studies have shown that attachment-related avoidance is related to cognitive distancing (Caldwell & Shaver, 2012; Holmberg et al., 2011) and suppression of emotional reactions (Maunder et al., 2006). Possibly, individuals with higher avoidance may deal with the pandemic, and associated distress, by distancing themselves from thoughts and concerns regarding the pandemic, by minimizing the dangers involved, and thus weaken their motivation to act in accordance with safety guidelines. Previous results showing that individuals with higher attachment avoidance report less precautionary actions (Lozano & Fraley, 2021), lower emotional distress (Moccia et al., 2020), as well as our findings linking avoidance to less knowledge about the safety guidelines (*r* = −.11, *p* < .01), speak in favor of this notion. Hence, the reluctance of highly avoidant people to act in accordance with the guidelines could be due to a combination of lower social trust and denial of COVID-19 threats, a combination which might also explain why social trust did not fully mediate the relationship between attachment-related avoidance and adherence.

### Attachment anxiety, negative emotions, and adherence

The experience of negative emotions was one of the strongest predictors of adherence to the authorities’ guidelines. Negative emotions could be a consequence of suffering from COVID-19, being in a risk-group (Margraf et al., 2020), or having relatives who are (Mazza et al., 2021). These are factors that should make one more inclined to adhere to the guidelines (Clark et al., 2020; Margraf et al., 2020). Negative emotions were also related to both attachment anxiety and trait anxiety. Interestingly though, despite the fact that attachment anxiety was positively related to the experience of negative emotions—which again were associated with higher degree of adherence to the guidelines—people higher in attachment anxiety were *less* (not more) inclined to follow the guidelines. They thus failed to act in a way that would remove a possible cause of their distress. This is in line with previous research indicating that despite highly attachment-anxious people reacting faster to threats (Ein-dor et al., 2011a), they suffer from more distress (Moccia et al., 2020) and turn to worrying and rumination rather than effective coping strategies (Caldwell & Shaver, 2012), which might further exacerbate negative emotions without leading to effective protective action.

Note also that trait anxiety was not related to adherence, implying that it is not anxiety in general that has an impact on adherence, but attachment-related anxiety. The guidelines explicitly ask people to maintain distance from each other, and to a large degree they encourage people to stay at home, away from friends and families. This is in stark contrast to the hyperactivation strategies involved in anxious attachment (Mikulincer & Shaver, 2016), whereby highly attachment-anxious people instead wish to seek proximity to others. To follow up on this idea, we examined post hoc the correlations between the specific guidelines at the item-level and attachment anxiety to see which items were most closely related to attachment anxiety. The three guidelines that stood out were all related to avoiding social contact (maintaining physical distance to others [*r* = −.14, *p* < .001], avoiding public transportation [*r* = −.17, *p* < .001], and avoiding restaurants and bars [*r* = −.11, *p* = .006]; for a full presentation, see online materials: https://osf.io/rac6k). Thus, the lower adherence associated with anxious attachment appears to be due to struggles associated with being alone (Mikulincer et al., 2021), especially in a threatening situation. It should also be noted that these results withstood control for pertinent covariates such as age, socioeconomic background, and trait anxiety. This marked propensity among highly attachment-anxious people to seek solace in social situations, despite other humans being the source of threat, can be contrasted with their unwillingness to trust non-social solutions to threats such as artificial intelligence (Gillath et al., 2020).

### Methodological considerations and future directions

The present study yielded novel findings regarding attachment and behavior related to risk and health, and how attachment representations may extend beyond close relationships. The results also add to prior research on attachment and emotional reactions in times of stress. However, the study had some important limitations. First, all participants were recruited through convenience sampling on social media, and there was an overrepresentation of women and people with higher education. Because the study was voluntary, there is a risk that participants were more interested in COVID-19 issues than people in general, which might also make them more inclined to act in accordance with the guidelines. This could explain the rather high mean values for adherence (5.72 on a 7-point scale). Notably, this would make variation on the outcome variable smaller and hence could increase the risk of not detecting effects or attenuating the size of true effects. Moreover, people with a lower degree of trust might have been less inclined to partake in the study. To mitigate some of these risks, we also collected participants through the web platform studentkaninen.se to target specifically male participants and reach out to people who might not be on the same social media network.

Additionally, the associations among negative emotions, attachment anxiety, and adherence should be interpreted with caution. Because we did not ask about negative emotions specifically in relation to COVID-19, nor included a control group who did not experience the pandemic, we cannot ascertain whether the higher degree of negative emotions among individuals with higher attachment anxiety is due to COVID-19 or to other factors. However, previous studies do indicate that attachment anxiety specifically is related to more pandemic-related distress (Mazza et al., 2021; Moccia et al., 2020). Similarly, because we did not ask specifically about coping strategies and intention to turn to other people during the pandemic, we cannot know whether these variables played a role in mediating the association between attachment-related anxiety and defiance of guidelines. We also did not ask about relational status of the participants, for example, whether they lived alone or not. Possibly, this could impact the relations among attachment anxiety, negative emotions, and adherence, as being alone in itself is related with more distress among individuals higher in attachment anxiety. We therefore stress that our conclusions regarding attachment orientations, emotional reactions, and adherence are *interpretations* of the data that require further research substantiation.

Also because the study was based entirely on cross-sectional data, causal direction could not be determined. Finally, because the data were based on self-reports, social desirability concerns might have biased people to overestimate their adherence to the guidelines. Again, however, this would decrease variation on the outcome variable and hence attenuate effect sizes. Additionally, other factors related to both attachment orientations and adherence that were not controlled for in our study, such as poor health, working constraints that prevent adherence to the guidelines, or parental responsibilities of leaving children at school, could have an impact on the results. We also did not include questions regarding race/ethnicity, sexual orientation, or disabilities and can thus draw no conclusions on how these attributes may have affected the results. We encourage future researchers to employ longitudinal designs and use more representative samples to further investigate the effects of attachment orientations on adherence.

To conclude, both attachment anxiety and avoidance were negatively associated with adherence to guidelines designed to prevent catching and spreading COVID-19. The finding for attachment anxiety is especially noteworthy because people who scored higher on that variable also reported a greater incidence of negative emotions, which itself was positively related to greater adherence to the guidelines. In other words, despite participants higher in attachment anxiety typically experiencing markedly negative emotions, they followed the public health agency’s recommendations to a lesser extent. This suggests how attachment anxiety (i.e., hyperactivation) can work in real-life settings and have emotional and behavioral consequences outside close interpersonal relationships.

## Appendix

Adherence to Authorities’ Guidelines, Items.

**Table:**

	Swedish original	English translation
1	Stannat hemma vid lättare förkylningssymtom	Stayed at home if you felt unwell
2	Tvättat händerna minst 20 sekunder efter att ha varit bland andra människor	Washed your hands for at least 20 seconds after you spent time among other people
3	Hållit minst 1,5 m avstånd till andra människor i offentliga miljöer, exempelvis mataffären eller lokaltrafiken	Kept at least 1.5 m distance to other people in public places, such as the grocery store or on public transportation
4	Undvikit att åka med offentliga färdmedel	Avoided traveling with public transportation
5	Undvikit fester, kalas, bröllop, begravningar, eller andra liknande sammanhang med många människor	Avoided parties, weddings, funerals or other similar contexts with large gatherings of people
6	Undvikit att gå på restaurang eller barer	Avoided visiting restaurants or bars
7	Undvikit resor inom sverige	Avoided traveling within Sweden

As part of IARR’s encouragement of open research practices, the author(s) have provided the following information: This research was pre-registered. The aspects of the research that were pre-registered were the hypothesis and the data-analysis plan. The registration was submitted to: https://osf.io/84796/. The data and materials used in the research can be obtained at https://osf.io/rac6k/or by emailing: joel.gruneau.brulin@psychology.su.se.
